# Causal Effects of Air Pollution, Noise, and Shift Work on Unstable Angina and Myocardial Infarction: A Mendelian Randomization Study

**DOI:** 10.3390/toxics13010021

**Published:** 2024-12-28

**Authors:** Qiye Ma, Lin Chen, Hao Xu, Yiru Weng

**Affiliations:** 1 Department of Emergency, Ningbo Ninth Hospital, Ningbo 315000, China; 2 Department of Epidemiology and Biostatistics, West China School of Public Health and West China Fourth Hospital, Sichuan University, Chengdu 610041, China; 3 Intensive Careful Unit, The Affiliated Lihuili Hospital of Ningbo University, Ningbo 315040, China

**Keywords:** unstable angina, myocardial infarction, shift work, air pollution, Mendelian randomization

## Abstract

Cardiovascular disease continues to be a major contributor to global morbidity and mortality, with environmental and occupational factors such as air pollution, noise, and shift work increasingly recognized as potential contributors. Using a two-sample Mendelian randomization (MR) approach, this study investigates the causal relationships of these risk factors with the risks of unstable angina (UA) and myocardial infarction (MI). Leveraging single nucleotide polymorphisms (SNPs) as genetic instruments, a comprehensive MR study was used to assess the causal influence of four major air pollutants (PM_2.5_, PM_10_, NO_2_, and NO_x_), noise, and shift work on unstable angina and myocardial infarction. Summary statistics were derived from large genome-wide association studies (GWASs) from the UK Biobank and the FinnGen consortium (Helsinki, Finland), with replication using an independent GWAS data source for myocardial infarction. The inverse-variance weighted (IVW) approach demonstrated a significant positive correlation between shift work and the increased risk of both unstable angina (OR with 95% CI: 1.62 [1.12–2.33], *p* = 0.010) and myocardial infarction (OR with 95% CI: 1.46 [1.00–2.14], *p* = 0.052). MR-PRESSO analysis identified outliers, and after correction, the association between shift work and myocardial infarction strengthened (OR with 95% CI: 1.58 [1.11–2.27], *p* = 0.017). No notable causal associations were identified for air pollution or noise with either outcome. The replication of myocardial infarction findings using independent data supported a possible causal link between shift work and myocardial infarction (OR with 95% CI: 1.41 [1.08–1.84], *p* = 0.012). These results provide novel evidence supporting shift work as a likely causal risk factor for unstable angina and myocardial infarction, underscoring the need for targeted public health strategies to mitigate its cardiovascular impact. However, further investigation is necessary to elucidate the role of air pollution and noise in cardiovascular outcomes.

## 1. Introduction

Acute coronary syndrome is one of the most prevalent and life-threatening conditions frequently encountered in emergency and critical care settings, encompassing unstable angina (UA) and myocardial infarction (MI) [1,2]. The pathophysiology of acute coronary syndrome is primarily attributed to the acute occlusion of coronary arteries, which results in a compromised blood supply to the myocardium and can lead to life-threatening complications, such as heart failure and sudden cardiac death [3]. Recent advances in interventional cardiology, particularly in the areas of early reperfusion techniques and percutaneous coronary interventions, have shown promise in improving patient outcomes. However, despite these advancements, acute coronary syndrome continues to pose significant challenges to global health [4,5]. The persistent impact of acute coronary syndrome on healthcare resources necessitates more research into effective prevention and management strategies to mitigate its burden on patients and healthcare systems.

Among the various modifiable risk factors for cardiovascular diseases, increasing attention is being paid to air pollution, environmental noise, and shift work due to their widespread prevalence and documented adverse health effects. Air pollution, primarily characterized by particulate matter, has been closely linked to cardiovascular diseases [6]. For instance, studies have shown that short-term exposure to air pollutants substantially raises the risk of hospitalization for non-ST-segment elevation acute coronary syndrome, particularly in individuals with unstable angina and hypertension [7,8]. In a retrospective analysis of 8737 patients with unstable angina, Zhang et al. identified a significant association between heightened exposure to PM_10_ and carbon monoxide and an elevated risk of hospital readmission due to heart failure. Specifically, the study highlighted a marked increase in risk when PM_10_ concentrations exceeded 112.5 µg/m^3^ and carbon monoxide levels surpassed 37.5 µg/m^3^ [9]. In addition to air pollution, environmental noise has emerged as a significant health concern. Chronic exposure to noise pollution is known to lead to chronic stress responses and elevated blood pressure, triggering inflammation of blood vessels and raising the risk of acute coronary syndrome [10]. A nationwide cohort study conducted in Switzerland reported that individuals exposed to both air pollution and noise experienced elevated mortality rates due to myocardial infarction [11]. More importantly, Vienneau et al. found that the detrimental effects of road traffic and rail noise on cardiovascular diseases began at exposure levels lower than the thresholds recommended by the World Health Organization [12]. Shift work, which disrupts biological rhythms, is another modifiable risk factor linked to adverse cardiovascular outcomes [13]. The relationship between shift work and cardiovascular disease has also received more and more attention from researchers, with evidence suggesting that shift work contributes to myocardial infarction risk through pathways involving hypertension, dyslipidemia, and metabolic disorders [14,15]. However, establishing a definitive causal relationship between these environmental exposures and cardiovascular outcomes poses substantial challenges. The potential for confounding variables and the risk of reverse causation inherent in traditional observational studies complicate the interpretation of these associations [16]. To advance our understanding of these relationships, it is imperative to employ more rigorous research methodologies, such as Mendelian randomization (MR).

Mendelian randomization offers a promising approach to addressing the challenges associated with establishing causal relationships in observational studies. Conceptually similar to a randomized controlled trial, MR utilizes genetic variants as instrumental variables (IVs), with randomization occurring at the point of zygote formation. This inherent design feature enhances the robustness of MR against reverse causation and minimizes the influence of confounding factors, making it a more reliable alternative to traditional observational research methodologies [17]. The purpose of this study is to examine the causal relationships between air pollution, environmental noise, and shift work, and the risk of UA and MI, using a two-sample MR design. By leveraging genetic instruments, this study provides novel insights into modifiable risk factors for cardiovascular disease.

## 2. Materials and Methods

### 2.1. Study Design

**Figure 1:**
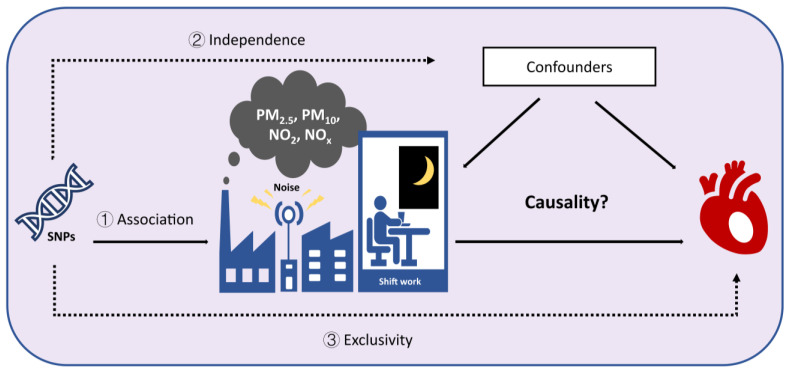
Summary chart of the main ideas of the paper.

**Figure 2:**
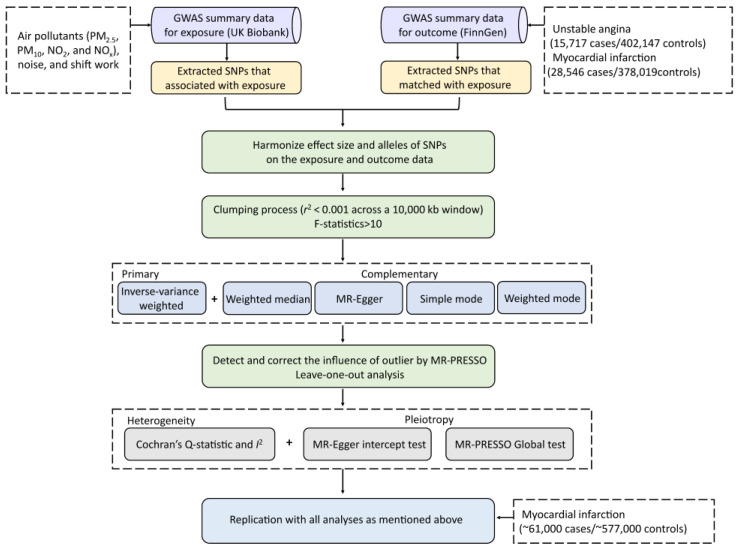
Overall study design and workflow.

The two-sample MR design relies on three fundamental principles (Figure 1) [18]. First of all, the IVs should exhibit a robust correlation with the exposure, ensuring it effectively represents the exposure being studied. Second, the IVs must be independent of any confounding factors that could affect the association between the exposure and the outcome, reducing potential bias. Third, the IVs must have an influence on the outcome exclusively through the exposure of interest, without affecting the outcome through alternative pathways. This ensures that the causal effect observed is directly mediated by the exposure, providing a clearer basis for causal inference. The overall study design and workflow for this comprehensive two-sample MR are illustrated in Figure 2. It is crucial to highlight that this study conformed to the reporting standards specified in the STROBE-MR (strengthening the reporting of observational studies in epidemiology using Mendelian randomization) framework [19]. The checklist for STROBE-MR can be found in the Supplementary Materials.

### 2.2. Data Source

Detailed information about each dataset used in the analysis is presented in Table 1. All GWAS summary data used in this two-sample MR study were sourced from the project website and were publicly available. In the original studies, informed consent was obtained from all participants. Consequently, no further ethical approval or additional consent is required.

### 2.3. Exposure GWAS(s)

In this study, we employed the most recent and largest GWAS summary data from the UK Biobank, covering four major air pollutants: PM_2.5_, PM_10_, NO_2_, and NO_x_. Particulate matter (PM) was categorized into PM_2.5_, consisting of particles with a diameter of 2.5 μm or less, and PM_10_, which encompasses particles no more than 10 μm in size. NO_x_, a term referring to nitrogen oxides (including NO and NO_2_), is primarily produced by fuel combustion and has varying levels of toxicity [20]. The UK Biobank is a groundbreaking, large-scale health research resource designed to advance the understanding of how genetic, environmental, and lifestyle factors influence human health. With data from approximately 500,000 participants across the United Kingdom, the Biobank has become an invaluable asset for studying the determinants of a wide range of diseases, including cardiovascular conditions, cancer, diabetes, and mental health disorders [21]. The land-use regression (LUR) model was adopted to predict pollutant concentrations. This model integrates environmental and geographic factors, including population density, land use, traffic patterns, and proximity to major roads or industrial areas. It has demonstrated strong predictive performance, achieving cross-validation R^2^ values of 77%, 88%, 87%, and 88% for PM_2.5_, PM_10_, NO_2_, and NO_x_, respectively, underscoring its reliability in capturing real-world pollutant exposure levels [22]. We also included GWAS summary statistics for noise exposure and shift work from the UK Biobank. Noise exposure was assessed using the “average 24-h sound level of noise pollution” metric, which provides a comprehensive measure of daily noise levels. This metric primarily reflects the general ambient noise levels in the living environments of the study participants. Shift work encompasses a broad range of schedules, including regular evening or night shifts, rotating shifts, split shifts, on-call or casual shifts, 24-h shifts, irregular schedules, and other non-standard working hours. While definitions may vary slightly across sources, shift work is generally defined as any work schedule that deviates from the conventional 9 am to 5 pm timeframe for an individual’s primary job [23].

### 2.4. Outcome GWAS(s)

The primary outcomes of this two-sample MR study were unstable angina and myocardial infarction. We acquired GWAS summary-level statistics for these outcomes from the FinnGen consortium R11 release data (FinnGen R11 release. https://r11.finngen.fi/ (Accessed on 10 July 2024)). The FinnGen study is an ongoing project, enriched for disease end points, that seeks to generate genomic data connected to national health registry records for 500,000 Finnish individuals [24]. The endpoints “Unstable angina pectoris” and “Myocardial infarction, strict” were used in this study, and the following variables were adjusted during the analysis: age, gender, genotyping batch, and the first 10 main components. To validate the reliability of our findings, we also used GWAS summary statistics of myocardial infarction from a meta-analysis performed by Hartiala et al., which included approximately 61,000 myocardial infarction patients and 577,000 healthy controls, making it the largest dataset available to date [25].

### 2.5. Sample Overlap Assessment

In two-sample MR studies, it is essential that the GWAS summary statistics for both exposure and outcome are derived from independent samples. Significant overlap between these samples can increase the risk of type 1 errors in causal inference [26]. MR results are considered more reliable when sample overlap is minimal, typically less than 10% [27]. To evaluate this, an online tool (Bias and Type 1 error rate for Mendelian randomization with sample overlap [https://sb452.shinyapps.io/overlap/ (accessed on 22 October 2024)]) can be used. In our primary analysis, the GWAS summary statistics for exposure were sourced from the UK Biobank, while those for the outcome were obtained from the FinnGen study, resulting in an overlap rate of 0%, which reduces the potential for Winner’s curse bias in our causal estimates.

### 2.6. Genetic Instruments Selection

For PM_2.5_, NO_2_, and NO_x_, independent single nucleotide polymorphisms (SNPs) reaching genome-wide significance were selected as IVs. These SNPs were chosen according to their genome-wide significance (*p* < 5 × 10^−8^) and their absence of linkage disequilibrium (LD) with other genetic variants ($r^{2}$ < 0.001 within the LD distance of ±5000 kb) from the original GWAS. For the remaining exposure traits (PM_10_, noise exposure, and shift work), a more lenient threshold of *p* < 5 × 10^−6^ was applied to ensure sufficient IVs, followed by LD pruning (*r*^2^  <  0.001 across a window of 10,000 kb). A harmonization process was subsequently performed to verify forward strand alleles and apply allele frequencies for palindromic variants as part of the quality control. The reliability of the MR results was contingent upon the robustness of the IVs. To evaluate the strength of the instruments, the Cragg–Donald F-statistic was calculated based on the formula $F=\frac{\left(N-2\right)R^{2}}{1-R^{2}}$, where $R^{2}$ represents the percentage of variance explained by the exposure, and *N* refers to the corresponding sample size [28]. In this study, $R^{2}$ for each IV was derived using the formula $R^{2}=\sum 2\times {\hat{\beta }}^{2}\times \mathrm{MAF}\times \left(1-\mathrm{MAF}\right)$, where $\hat{\beta }$ represents the estimated IV-exposure association and MAF refers to the minor allele frequency associated with each genetic variant.

### 2.7. Statistical Analysis

This study employed five analytical approaches within the two-sample MR framework, comprising inverse-variance weighted (IVW), weighted median, and MR-Egger regression, along with weighted mode and simple mode approaches. The primary approach for causal inference was the IVW approach, which estimates the causal effect of each genetic instrument via the Wald ratio. These individual estimates are then pooled using either a fixed-effect or random-effect model to provide a reliable causal estimate under the assumption that all SNPs are valid instruments [29]. The weighted median method was applied as the supplementary method, which can provide reliable estimates even if as much as 50 percent of the IVs are invalid [30]. MR-Egger regression relaxes the third IV assumption by including an intercept term, which can indicate pleiotropy if the intercept is significantly different from zero [31]. In addition, mode-based approaches (both weighted and simple) offer consistency in results, even when a majority of SNPs are invalid [32]. To further control for potential horizontal pleiotropy, the MR-PRESSO (Mendelian randomization pleiotropy residual sum and outlier) was utilized to detect the effects of outlier SNPs [33]. Leave-one-out analyses were also performed by removing SNPs one by one to verify that no single SNP was driving the results. If excluding a specific SNP significantly changes the MR estimate, this implies that this IV may have a direct influence on the outcome, thus violating the third core assumption of MR. Additionally, to evaluate the heterogeneity among the selected IVs, Cochran’s Q-statistic with *I*^2^ was computed, and the existence of heterogeneity was signified by a *p*-value of below 0.05 [34].

We regarded the causal association as reliable if the following three conditions were met: (1) the MR–IVW estimate for exposure showed a significant association with the outcome; (2) the direction of the IVW estimate remained directionally consistent across all sensitivity analyses; and (3) there was minimal evidence of horizontal pleiotropy, as indicated by the MR-PRESSO global test or MR-Egger intercept. To account for multiple testing across six exposures and two outcomes, a Bonferroni-corrected two-sided *p*-value of <0.004 was deemed strong evidence, while *p*-values < 0.05 were considered suggestive evidence. All statistical analyses in this study were conducted using R software (version 4.2.2, R Foundation for Statistical Computing, Vienna, Austria) with the implementation of the “TwoSampleMR” package (version 0.6.8), the “MRPRESSO” package (version 1.0), and the “MendelianRandomization” package (version 0.7.0). Visualization of results, including forest plots and leave-one-out plots, was achieved using the “ggplot2” package (version 3.5.1) and the “forestplot” package (version 2.0.1).

## 3. Results

### 3.1. Characteristics of Genetic Instruments

The characteristics of the IVs identified for each exposure are summarized in Table 1, with more details provided in Table S1. Notably, of the eight independent IVs for PM_2.5_, one SNP was unavailable in the outcome GWAS. Consequently, the remaining seven SNPs were used as IVs, each showing an F-statistic greater than 10. Similarly, after screening, 28 SNPs for PM_10_ and 35 SNPs for shift work were retained for the MR analysis. The estimated F-Statistics for all IVs across the exposures varied from 15 to 67, suggesting that weak instrument bias was minimized.

### 3.2. Causal Effects of Air Pollution, Noise, and Shift Work on Unstable Angina

**Figure 3:**
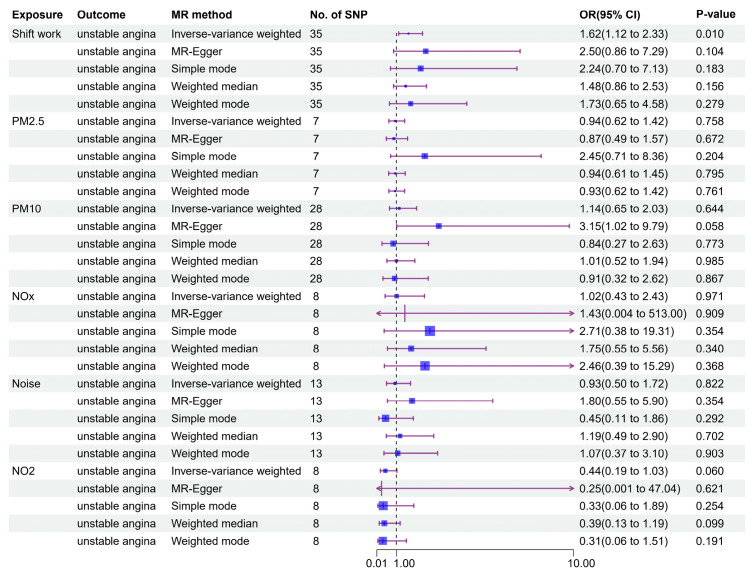
Causal effects of air pollution, noise, and shift work on unstable angina.

As shown in Figure 3, a significant correlation between shift work and an increasing risk of unstable angina was revealed by the IVW approach (OR [95 %CI]: 1.62 [1.12~2.33], *p* = 0.010). Directionally consistent results were also found in the other four complementary methods, although they were not significant (all *p*-values > 0.05). Notably, no heterogeneity was observed in the IVW results, as indicated by a *p*-value above 0.05 from Cochran’s Q test (Table S2). Additionally, the MR-Egger regression intercept was near zero, suggesting that horizontal pleiotropy did not materially impact the MR findings (Table S3). The MR-PRESSO analysis corroborated the IVW results, showing similar estimates (OR [95 %CI]: 1.62 [1.14~2.29], *p* = 0.011, Table 2). The robustness of the overall findings was verified by the leave-one-out analysis because no individual SNP unduly influenced the results (Figure S1).

In contrast, the IVW approach found no causal link between unstable angina and other exposures, as shown using the IVW approach (PM_2.5_: 0.94 (0.62–1.42), *p* = 0.758; PM_10_: 1.14 (0.65–2.03), *p* = 0.644; NO_2_: 0.44 (0.19–1.03), *p* = 0.060; NO_x_: 1.02 (0.43–2.43), *p* = 0.971; Noise: 0.93 (0.50–1.72), *p* = 0.822, Figure 3).

### 3.3. Causal Effects of Air Pollution, Noise, and Shift Work on Myocardial Infarction

**Figure 4:**
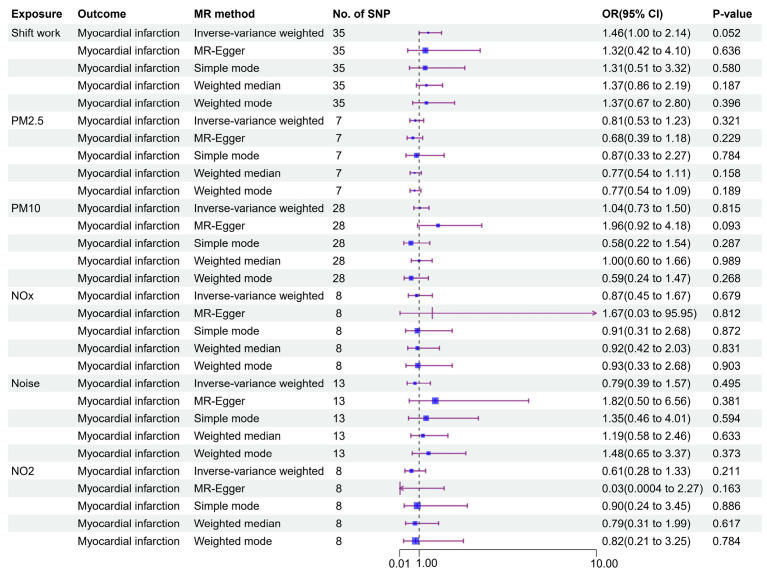
Causal effects of air pollution, noise, and shift work on myocardial infarction.

To some extent, heterogeneity (*I*^2^ = 43.27%) was found for the correlation between shift work and myocardial infarction, so the IVW method using the random-effects model was adopted as the main method for calculating MR estimates (Table S2). As shown in Figure 4, there was a positive, albeit marginally significant, correlation of shift work with myocardial infarction risk (OR [95 %CI]: 1.46 [1.00~2.14], *p* = 0.052), with similar trends observed in the four additional MR methods. To further investigate potential biases, the MR-Egger intercept test was applied, but no obvious signal of pleiotropic effects was revealed (Table S3). In MR-PRESSO, however, one SNP (rs61844343) was identified as the outlier (global test *p* = 0.005), which might have influenced the results (Table 2). After excluding this SNP, the MR-PRESSO method exhibited a stronger, statistically significant correlation of shift work with an elevated myocardial infarction risk (OR [95% CI]: 1.58 [1.11–2.27], *p* = 0.017, Table 2). The leave-one-out analysis corroborated the findings from the MR-PRESSO method, further supporting the robustness of the causal estimates (Figure S2). To guarantee the consistency and dependability of our results, we replicated the identified associations using GWAS summary statistics of myocardial infarction from Hartiala et al. As shown in Table 3, this replication supported a possible causal link of shift work with myocardial infarction risk (OR [95 %CI]: 1.41 [1.08–1.84], *p* = 0.012), with no obvious signals of pleiotropic effects or heterogeneity detected in the analysis, further validating the original findings.

## 4. Discussion

Using the largest GWAS summary data conducted to date, this two-sample MR was utilized to assess the potential causal associations of air pollution, noise, and shift work on the risks of unstable angina and myocardial infarction. Our findings suggest that shift work may be causally correlated with an elevated risk of both unstable angina and myocardial infarction, underscoring the detrimental impact of irregular work schedules on cardiovascular health. However, no obvious causal effects were detected for air pollution or noise exposure. These findings carry significant public health implications, particularly for populations disproportionately affected by shift work. The results suggest an urgent need for targeted preventive strategies aimed at mitigating cardiovascular risks in shift workers, such as workplace interventions, health monitoring programs, and policies promoting healthier work environments. By addressing the unique cardiovascular challenges faced by individuals engaged in shift work, these measures could help reduce the incidence of unstable angina and myocardial infarction and improve long-term cardiovascular outcomes.

The results from the present study are in line with the majority of previous observational studies, but go further by demonstrating the possible causal effects of shift work on the risks of unstable angina and myocardial infarction. Indeed, lots of observational studies regarding shift work and adverse cardiovascular outcomes have been explored. For instance, a large-scale cohort study by Vyas et al. analyzed the association of shift work with vascular events, including myocardial infarction. Their meta-analysis, which included over two million participants, found that working in shifts was correlated with a 23% increased risk of myocardial infarction and other cardiovascular outcomes [23]. In another systematic review of observational studies, shift workers were observed to present a heightened risk of angina pectoris and myocardial infarction, reinforcing concerns about the cardiovascular consequences of irregular work schedules [35]. Similarly, an umbrella review conducted by Wu et al., including eight eligible systematic reviews and meta-analyses, also identified strong indicative evidence for associations of shift work with myocardial infarction (having ever vs. having never done shift work) [36]. However, given the low hierarchy of evidence from observational studies, the nature of the associations is still less than certain. For instance, the Kuopio ischemic heart disease risk factor study presented mixed results regarding the relationship of diverse categories of shift work with the 20-year incidence of myocardial infarction among individuals with and without preexisting ischemic heart disease [37].

Inconsistent with a previous MR study, our MR results, utilizing data from the hitherto largest GWAS, present more reliable evidence for the possible causal link between shift work and unstable angina and myocardial infarction [38]. Using the IVW approach, we found an obvious positive correlation between shift work and elevated risks of unstable angina and myocardial infarction. The uniformity of our findings across four additional MR analyses further supports the robustness of these associations. For the association between shift work and unstable angina, no heterogeneity or directional pleiotropy was observed in the study, and the MR-PRESSO analysis showed similar estimates to the IVW results. Although an SNP (rs61844343) was identified as an outlier for the association between shift work and myocardial infarction, the corrected estimate of MR-PRESSO for this outlier SNP strengthened the link between shift work and myocardial infarction, emphasizing the validity of our results. More importantly, replication using an independent GWAS dataset for myocardial infarction confirmed the reliability of our findings, establishing shift work as an important risk factor for cardiovascular diseases.

The mechanisms underlying the association of shift work and myocardial infarction are likely related to disrupted circadian rhythms, chronic increased stress, and metabolic dysregulation, as suggested in prior research. Disturbed sleep, a frequent complaint among night shift workers, has been identified as an independent risk factor for myocardial infarction [39]. This health concern arises from the mismatch between unconventional work hours and the body’s biological clock. A previous study highlighted that non-standard work schedules, especially those that include night or rotating shifts, can significantly disrupt the body’s natural circadian rhythm [40]. Such disruptions not only lead to chronic sleep deprivation, but also trigger a cascade of physiological changes, including altered circadian central nervous system activity, hormonal imbalances, and heightened cortisol levels [41,42]. These changes contribute to systemic inflammation and endothelial dysfunction, both of which are critical pathways in the development of cardiovascular diseases, including myocardial infarction [43]. Moreover, chronic stress, another common issue faced by shift workers, can induce a range of physiological and behavioral changes that are closely linked to cardiovascular disease [44]. Chronic stress can lead to hypertension by triggering the body’s stress response, which often involves elevated heart rate and vasoconstriction that subsequently increase blood pressure [45]. This continuous elevation in blood pressure contributes to the risk of cardiovascular diseases. In addition to these direct physiological effects, stress can indirectly influence cardiovascular health by promoting unhealthy behaviors. For instance, the Finnish public sector study, which included more than 50,000 individuals, found that smokers experiencing work-related stress were 50% more prone to smoking more than 20 cigarettes a day compared to individuals who did not report such stress [46]. The combination of hypertension and harmful habits amplifies the threat to cardiovascular health. Additionally, metabolic dysregulation, including insulin resistance, elevated triglyceride levels, and obesity, further exacerbates the risk, as shift work has been linked to poor dietary habits, physical inactivity, and abnormal melatonin levels [47]. Together, these mechanisms provide a plausible biological explanation for the increased incidence of myocardial infarction observed in individuals with irregular work patterns.

The lack of significant causal associations for air pollution and noise exposure in this study was unexpected given the well-recognized links of these environmental factors with cardiovascular disease in observational studies. Air pollution, specifically fine particulate matter (PM_2.5_), has been shown to contribute to the development and progression of cardiovascular and cerebrovascular diseases, such as stroke, coronary atherosclerosis, and myocardial infarction, through mechanisms involving oxidative stress, systemic inflammation, and endothelial dysfunction [48]. Similarly, exposure to noise pollution, especially chronic exposure to traffic and industrial noise, has been associated with increased risk of cardiovascular events by elevating blood pressure, promoting stress responses, and inducing vascular inflammation [49]. However, our MR analysis suggests that these exposures might not have a direct impact on the development of unstable angina and myocardial infarction.

One possible explanation for this divergence could be the inherent differences between observational studies and MR analyses [50,51]. Observational studies are prone to confounding factors that may overestimate the impact of environmental exposure on health outcomes. In contrast, MR, by using genetic variants as IVs, minimizes confounding and reverse causality, potentially providing a more accurate estimate of causal effects. It is possible that, while air pollution and noise are significant contributors to cardiovascular disease in the general population, their direct causal impact on specific outcomes like unstable angina and myocardial infarction may be smaller than previously thought or subject to additional moderating factors not accounted for in traditional observational studies. Another consideration is the possibility of measurement errors in the exposure data. Although the LUR model used in this study is a widely accepted method in epidemiological research, it may not fully capture individual variations in exposure, particularly in indoor environments or specific occupational settings [52]. Consequently, it is essential to highlight the potential benefits of incorporating environmental monitoring data in future studies, as this could significantly improve the accuracy and precision of exposure assessments in research on the health effects of air pollution. It is also worth considering that the effects of air pollution and noise might be mediated through pathways not captured by the genetic instruments used in this study. As a result, our findings do not completely rule out the potential association of air pollution and noise with these cardiovascular conditions.

This study has some advantages, including the implementation of a two-sample MR design, which minimizes bias due to reverse causation and confounding factors, and the use of large GWAS datasets, which enhances the statistical power to detect causal associations. More importantly, unlike several previous MR studies on air pollution and noise, this study adhered strictly to the MR study design principles, particularly by using independent GWAS datasets without sample overlap for causal inference. This approach enhances the reliability of the results by reducing the potential bias introduced by sample overlap, a common limitation in previous MR studies [53,54,55]. However, there are limitations that need to be addressed. First, although MR is robust against certain biases, it relies on the validity of instrumental variables, and weak instrument bias could affect the results, particularly for exposures like noise, where fewer genetic instruments were available. Second, directional pleiotropy arising from unrecognized confounding factors could distort the causal estimates. Nevertheless, we made efforts to minimize this bias. The consistency of the results across various ‘pleiotropy-robust’ methods, such as MR-PRESSO and MR-Egger regression, supports the credibility of our MR results. Lastly, the generalizability of the study may be limited, as the GWAS data used were predominantly derived from participants of European descent. This ethnic homogeneity may restrict the applicability of our findings to other populations, as genetic variations and environmental exposures can differ significantly across diverse ethnic groups. Future studies incorporating multiethnic cohorts are essential to validate our findings and ensure broader applicability, as well as to better understand potential genetic and environmental interactions in different populations.

## 5. Conclusions

In this study, we employed a two-sample Mendelian randomization design to investigate the causal relationship between shift work, air pollution, noise, and the risk of UA and MI. Our findings provide strong evidence that shift work is a possible causal risk factor for both UA and MI. These results underscore the need for targeted public health interventions to mitigate the cardiovascular risks associated with irregular work schedules. Further research is needed to explore the role of environmental factors, such as air pollution and noise, in cardiovascular disease and to better understand the underlying biological mechanisms through which shift work affects heart health.

## Figures and Tables

**Table 1:** Characteristics of selected GWASs in this study.

Trait	GWAS ID /Consortium /PMID	Ethnicity	Sample Size	Numbers of SNPs
Exposure				
PM_2.5_	ukb-b-10817	European	423,796	8
PM_10_	ukb-b-18469	European	423,796	29
NO_2_	ukb-b-9942	European	456,380	8
NO_x_	ukb-b-12417	European	456,380	8
Noise	ukb-b-19490	European	456,380	13
Shift work	ukb-b-1712	European	263,315	36
Outcome				
Unstable angina	FinnGen	European	15,717 cases/402,147 controls	-
Myocardial infarction	FinnGen	European	28,546 cases/378,019 controls	-
Myocardial infarction	33532862	European	~61,000 cases/~577,000 controls	-

**Table 2:** The MR estimates from the MR-PRESSO method.

Outcome	Exposure	Global Test *p*-Value	OR	95% CI	*p*-Value	Remove SNP
Unstable angina	PM_2.5_	0.611	0.936	0.617–1.421	0.768	0
	PM_10_	0.034	1.144	0.646–2.027	0.648	0
	NO_2_	0.464	0.445	0.195–1.011	0.094	0
	NO_x_	0.367	1.017	0.425–2.430	0.972	0
	Noise	0.666	0.932	0.538–1.614	0.805	0
	Shift work	0.655	1.616	1.140–2.291	0.011	0
Myocardial infarction	PM_2.5_	0.320	0.807	0.529–1.232	0.359	0
	PM_10_	0.619	1.044	0.740–1.474	0.807	0
	NO_2_	0.196	0.609	0.279–1.326	0.252	0
	NO_x_	0.930	0.871	0.579–1.311	0.530	0
	Noise	0.025	0.956	0.532–1.719	0.884	1
	Shift work	0.005	1.584	1.108–2.265	0.017	1

**Table 3:** Causal effects of air pollution, noise, and shift work on myocardial infarction in the validation samples.

Exposure	MR Method	No. of SNPs	OR	95% CI	*p*-Value	Heterogeneity		MR-Egger Intercept Test		MR-PRESSO Global Test
						Q	*p*-Value	Intercept	*p*-Value	*p*-Value
Shift work	Inverse-variance weighted	33	1.41	1.08–1.84	0.012	42.02	0.1106	−0.0079	0.259	0.121
	MR-Egger	33	2.41	0.93–6.26	0.081					
	Weighted median	33	1.36	0.96–1.93	0.086					
	Weighted mode	33	1.36	0.74–2.51	0.327					
	Simple mode	33	1.40	0.72–2.74	0.329					

## Data Availability

Data sharing is not relevant to this study, since no new data were generated or examined in this study. The primary sources of all data are presented in the “Data Source” section.

## Supplementary Materials

The following supporting information can be downloaded at: https://www.mdpi.com/article/10.3390/toxics13010021/s1, Figure S1: The leave-one-out analysis for unstable angina; Figure S2: The leave-one-out analysis for myocardial infarction; Table S1: Instrumental variables used in MR analysis; Table S2: The heterogeneity of instrumental variables; Table S3: Directional horizontal pleiotropy assessed by intercept term in MR Egger regression.
