# Supplementary material for: Causal Effects of Air Pollution, Noise, and Shift Work on Unstable Angina and Myocardial Infarction: A Mendelian Randomization Study
Source: Toxics. 2024 Dec 28;13(1):21. doi: 10.3390/toxics13010021 (PMC11768850; doi:10.3390/toxics13010021)
Supplement: Supplementary file 1 [file toxics-13-00021-s001.zip › STROBE-MR checklist.pdf]

Supplementary Material

STROBE-MR checklist of recommended items to address in reports of Mendelian randomization studies

Item No.	Section	Checklist item	Page No.	Relevant text from manuscript
1	TITLE and ABSTRACT	Indicate Mendelian randomization (MR) as the study's design in the title and/or the abstract if that is a main purpose of the study	1	Causal Effects of Air Pollution, Noise, and Shift Work on Unstable Angina and Myocardial Infarction: A Mendelian Randomization Study
**INTRODUCTION**				
2	Background	Explain the scientific background and rationale for the reported study. Is causality between exposure and outcome plausible? Justify why MR is a helpful method to address the study question.	3-4	Introduction, Paragraph 1-3
3	Objectives	State specific objectives clearly, including pre-specified causal hypotheses (if any). State that MR is a method that, under specific assumptions, intends to estimate causal effects	4	Introduction, Paragraph 4.
**METHODS**				
4	Study design and data sources	Present key elements of the study design early in the article. Consider including a table listing sources of data for all phases of the study. For each data source contributing to the analysis, describe the following:		

	a)	Setting: Describe the study design and the underlying population, if possible. Describe the setting, locations, and relevant dates, including periods of recruitment, exposure, follow-up, and data collection, when available.	4-5	Materials and Methods, Section “Study design"; Section “Data sources".
	b)	Participants: Give the eligibility criteria, and the sources and methods of selection of participants. Report the sample size, and whether any power or sample size calculations were carried out prior to the main analysis	5-6	Materials and Methods, Section “Data sources"; Table 1.
	c)	Describe measurement, quality control and selection of genetic variants	7	Materials and Methods, Section “Genetic instruments selection".
	d)	For each exposure, outcome, and other relevant variables, describe methods of assessment and diagnostic criteria for diseases	5-6	Materials and Methods, Section “Data sources"
	e)	Provide details of ethics committee approval and participant informed consent, if relevant	5	Materials and Methods, Section “Data sources".
5	Assumptions	Explicitly state the three core IV assumptions for the main analysis (relevance, independence and exclusion restriction) as well assumptions for any additional or sensitivity analysis	4	Materials and Methods, Section “Study design".
6	Statistical methods: main analysis	Describe statistical methods and statistics used		

	a)	Describe how quantitative variables were handled in the analyses (i.e., scale, units, model)	-	Not applicable to our study.
	b)	Describe how genetic variants were handled in the analyses and, if applicable, how their weights were selected	7	Materials and Methods, Section " Genetic instruments selection ".
	c)	Describe the MR estimator (e.g. two-stage least squares, Wald ratio) and related statistics. Detail the included covariates and, in case of two-sample MR, whether the same covariate set was used for adjustment in the two samples	7	Materials and Methods, Section “Statistical analysis".
	d)	Explain how missing data were addressed	—	Not applicable to our study.
	e)	If applicable, indicate how multiple testing was addressed	8	Materials and Methods, Section “Statistical analysis".
7	Assessment of assumptions	Describe any methods or prior knowledge used to assess the assumptions or justify their validity	7	Materials and Methods, Section " Genetic instruments selection ".

8	Sensitivity analyses and additional analyses	Describe any sensitivity analyses or additional analyses performed (e.g. comparison of effect estimates from different approaches, independent replication, bias analytic techniques, validation of instruments, simulations)	6-8	Materials and Methods, Section “Data sources"; Materials and Methods, Section “Statistical analysis ".
9	Software and pre registration			
	a)	Name statistical software and package(s), including version and settings used	8	Materials and Methods, Section "Statistical Analysis".
	b)	State whether the study protocol and details were pre-registered (as well as when and where)	—	No pre-registration.
**RESULTS**				
10	Descriptive data			
	a)	Report the numbers of individuals at each stage of included studies and reasons for exclusion. Consider use of a flow diagram	8	Results, Section "Characteristics of genetic instruments ".
	b)	Report summary statistics for phenotypic exposure(s), outcome(s), and other relevant variables (e.g. means, SDs, proportions)		
	c)	If the data sources include meta-analyses of previous studies, provide the assessments of heterogeneity across these studies	—	Not applicable to our study.
	d)	For two-sample MR:		

		i. Provide justification of the similarity of the genetic variant-exposure associations between the exposure and outcome samples	5-6	Materials and Methods, Section " Data sources ".
		ii. Provide information on the number of individuals who overlap between the exposure and outcome studies	6	Materials and Methods, Section " Sample overlap assessment".
11	Main results			
	a)	Report the associations between genetic variant and exposure, and between genetic variant and outcome, preferably on an interpretable scale	8	Results, Section "Characteristics of genetic instruments ".
	b)	Report MR estimates of the relationship between exposure and outcome, and the measures of uncertainty from the MR analysis, on an interpretable scale, such as odds ratio or relative risk per SD difference	8-10	Results, Sections " Causal effects of air pollution, noise, and shift work on unstable angina "; “Causal effects of air pollution, noise, and shift work on myocardial infarction”.
	c)	If relevant, consider translating estimates of relative risk into absolute risk for a meaningful time period	-	Not applicable to our study.
	d)	Consider plots to visualize results (e.g. forest plot, scatterplot of associations between genetic variants and outcome versus between genetic variants and exposure)	Figure 3, 4; Figure S1,S2	
12	Assessment of assumptions			
	a)	Report the assessment of the validity of the assumptions	8-9, supplementary tables	Results, Sections " Causal effects of air pollution, noise, and shift work on unstable angina ";

				“Causal effects of air pollution, noise, and shift work on myocardial infarction”.
	b)	Report any additional statistics (e.g., assessments of heterogeneity across genetic variants, such as I2, Q statistic or E-value)	8-9, supplementary tables	Results, Sections " Causal effects of air pollution, noise, and shift work on unstable angina "; “Causal effects of air pollution, noise, and shift work on myocardial infarction”.
13	Sensitivity analyses and additional analyses			
	a)	Use sensitivity analyses to assess the robustness of the main results to violations of the assumptions		Relevant information is given in the results section.
	b)	Report results from other sensitivity analyses (e.g., replication study with different dataset, analyses of subgroups, validation of instrument(s), simulations, etc)		To guarantee the consistency and dependability of our results, we replicated the identified associations using GWAS summary statistics of myocardial infarction from Hartiala et al.
	c)	Report any assessment of direction of causality (e.g., bidirectional MR)	-	Not applicable to our study.
	d)	When relevant, report and compare with estimates from non-MR analyses	10	Discussion, Paragraph 2.
	e)	Consider any additional plots to visualize results (e.g., leave-one-out analyses)		Figure S1,S2

DISCUSSION				
14	Key results	Summarize key results with reference to study objectives	13	Conclusion
15		Discuss limitations of the study, taking into account the validity of the MR assumptions, other sources of potential bias, and imprecision. Discuss both direction and magnitude of any potential bias, and any efforts to address them	13	Discussion, Paragraph 7.
16	Interpretation			
	a)	a) Give a cautious overall interpretation of results considering objectives and limitations Compare with results from other relevant studies.	10,11	Discussion, Paragraph 2,3.
	b)	b) Discuss underlying biological mechanisms that could be modelled by using the genetic variants to assess the relationship between the exposure and the outcome.	11,12	Discussion, Paragraph 4.
	c)	c) Discuss whether the results have clinical or policy relevance, and whether interventions could have the same size effect	10	Discussion, Paragraph 1.
17	Generalizability	Discuss the generalizability of the study results (a) to other populations, (b) across other exposure periods/timings, and (c) across other levels of exposure	13	Discussion, Paragraph 7.

**OTHER INFORMATION**				
18	Funding	Describe sources of funding and the role of funders in the present study and, if applicable, sources of funding for the databases and original study or studies on which the present study is based	14	Section “Funding”.
19	Data and data sharing	Provide the data used to perform all analyses or report where and how the data can be accessed, and reference these sources in the article. Provide the statistical code needed to reproduce the results in the article, or report whether the code is publicly accessible and if so, where	14	Section "Data Availability Statement".
20	Conflicts of Interest	All authors should declare all potential conflicts of interest	14	Section “Conflict of interest statement”.
