# Supplementary figures and images for: Causal Effects of Air Pollution, Noise, and Shift Work on Unstable Angina and Myocardial Infarction: A Mendelian Randomization Study
Source: Toxics. 2024 Dec 28;13(1):21. doi: 10.3390/toxics13010021 (PMC11768850; doi:10.3390/toxics13010021)

A

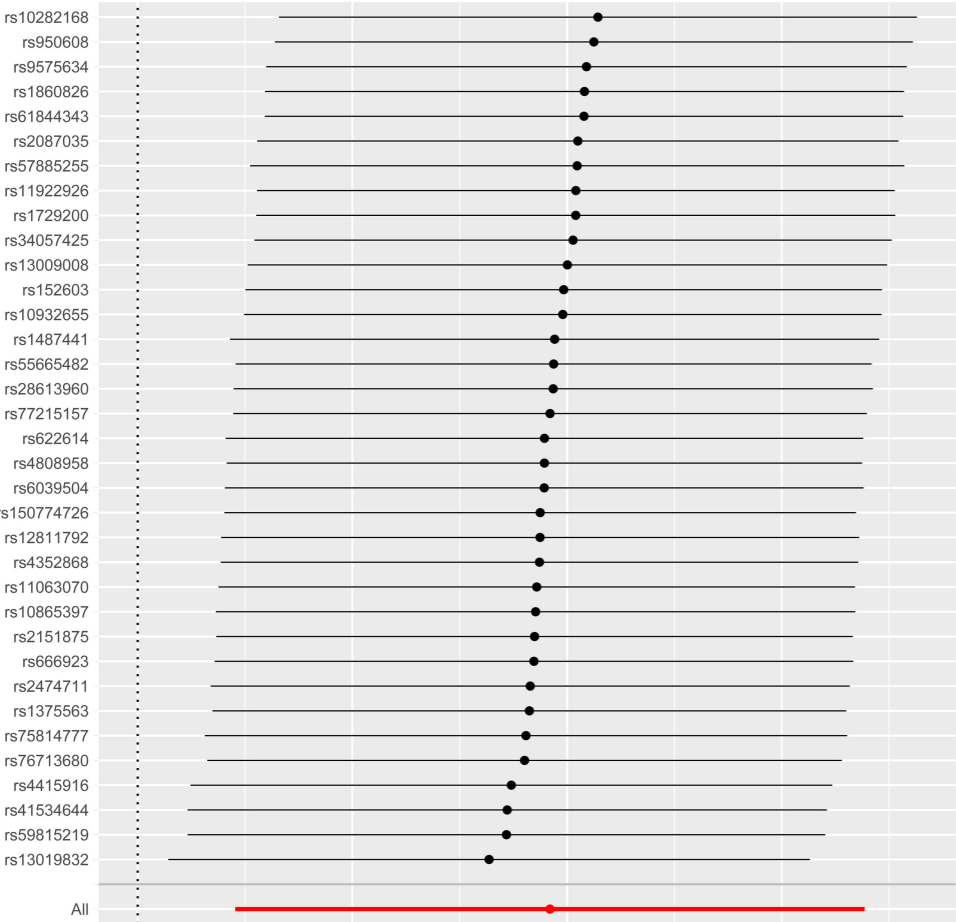

B

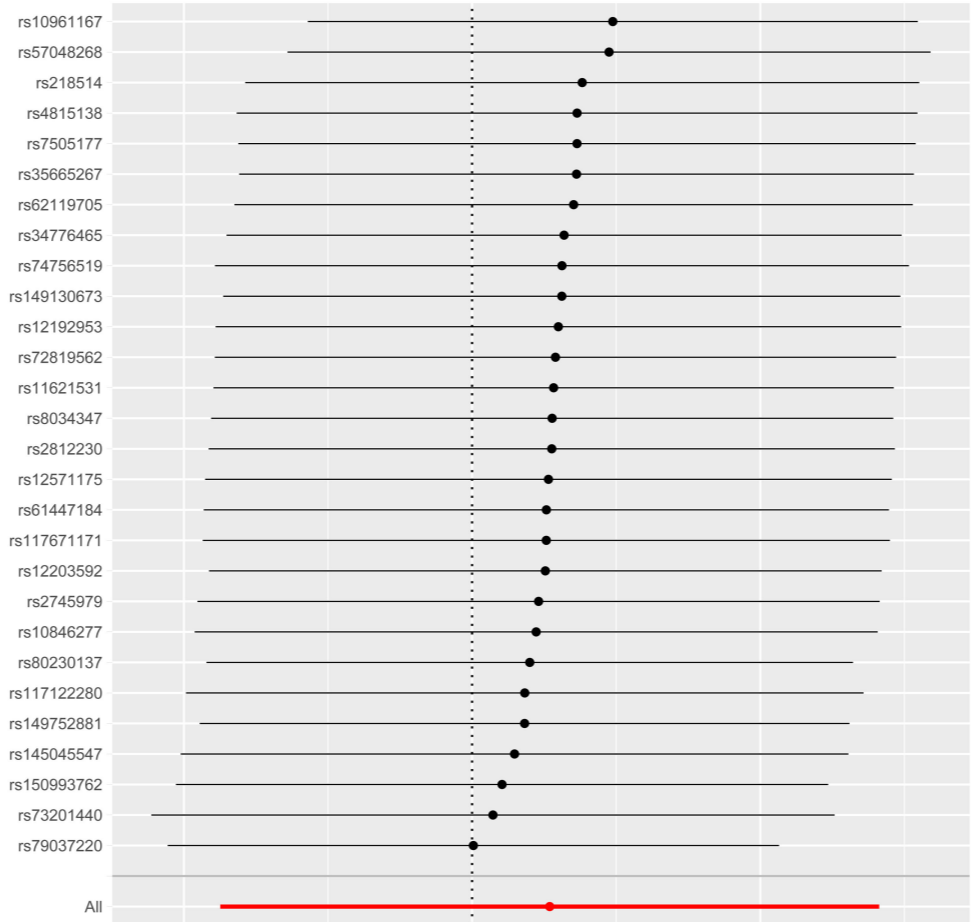

C

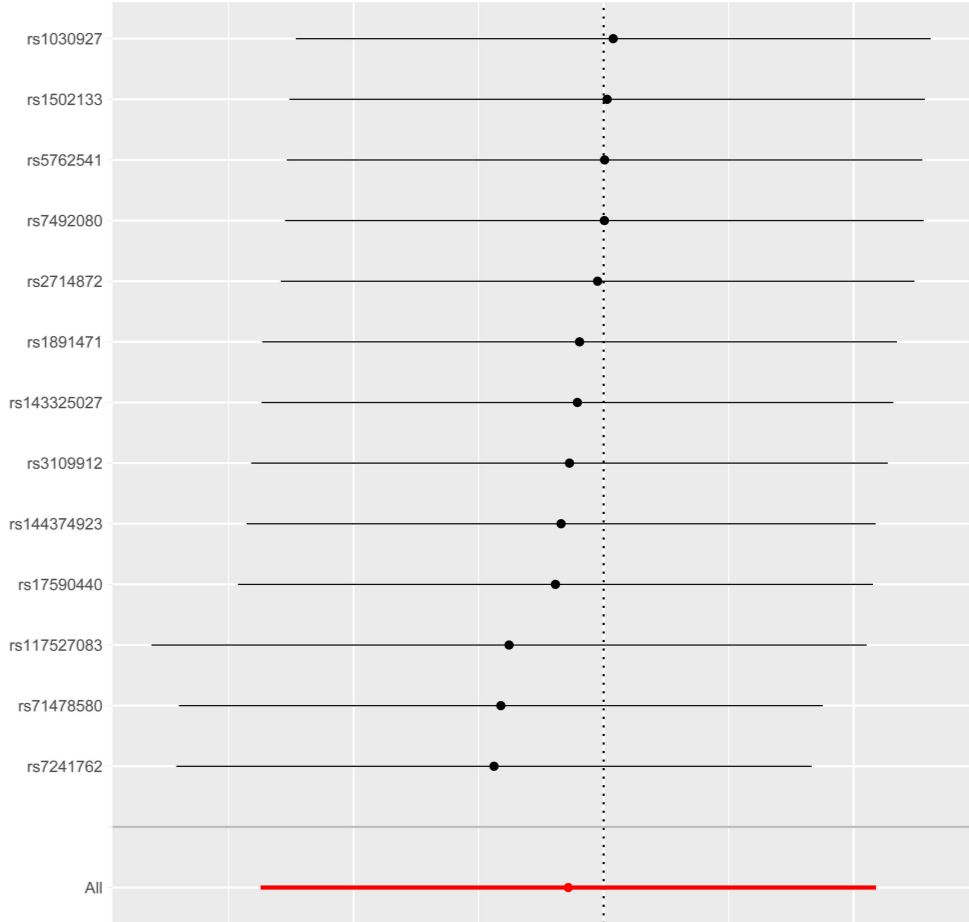

D

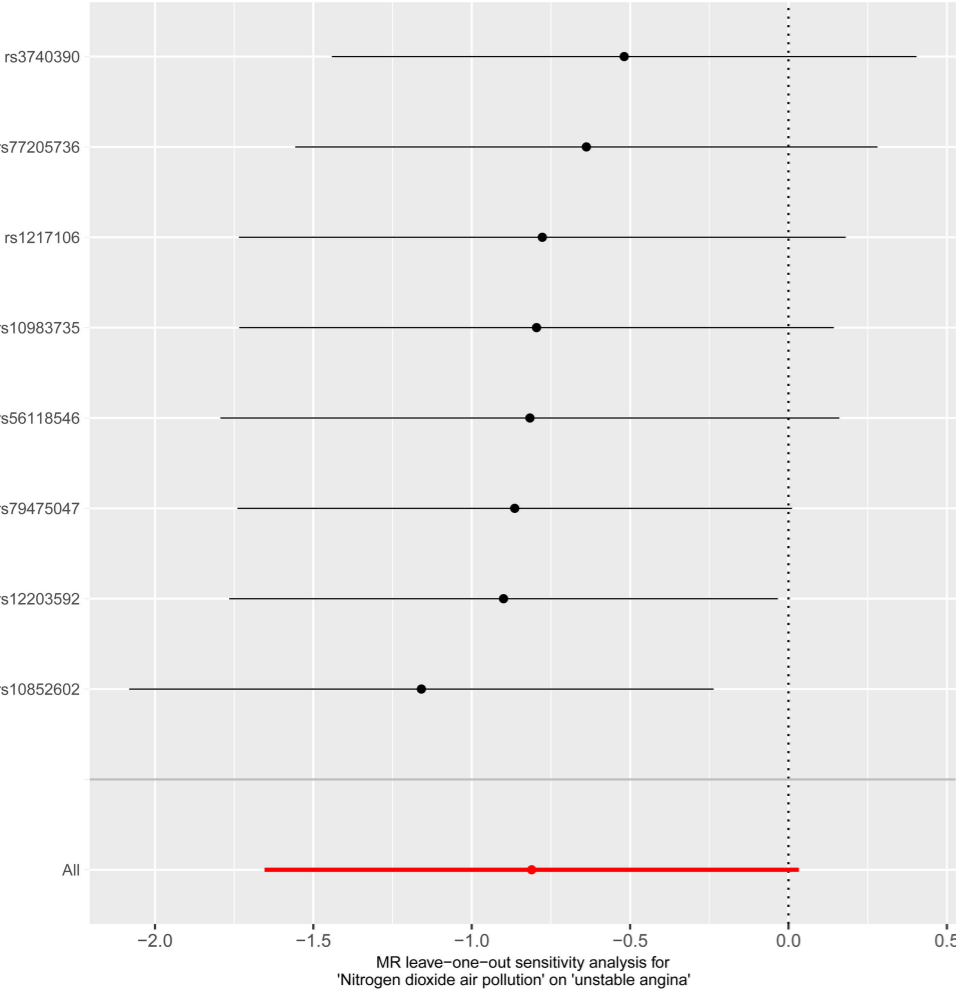

E

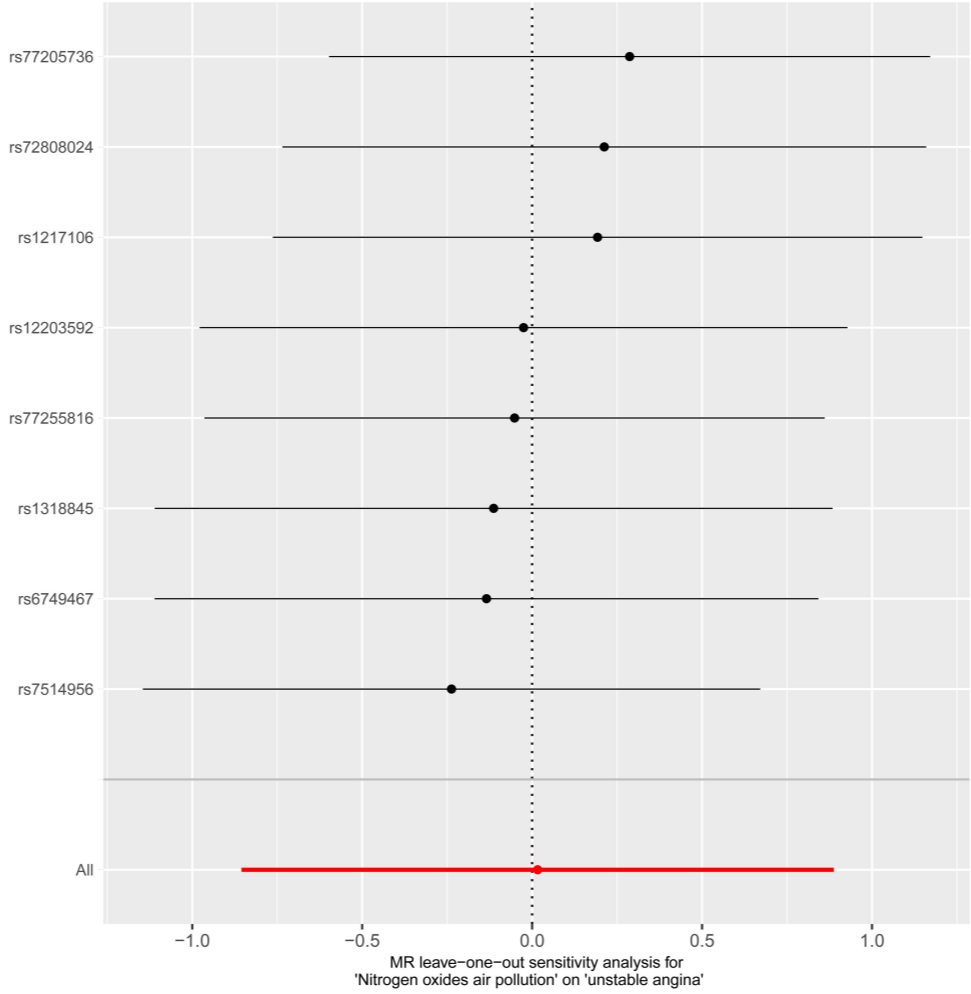

F

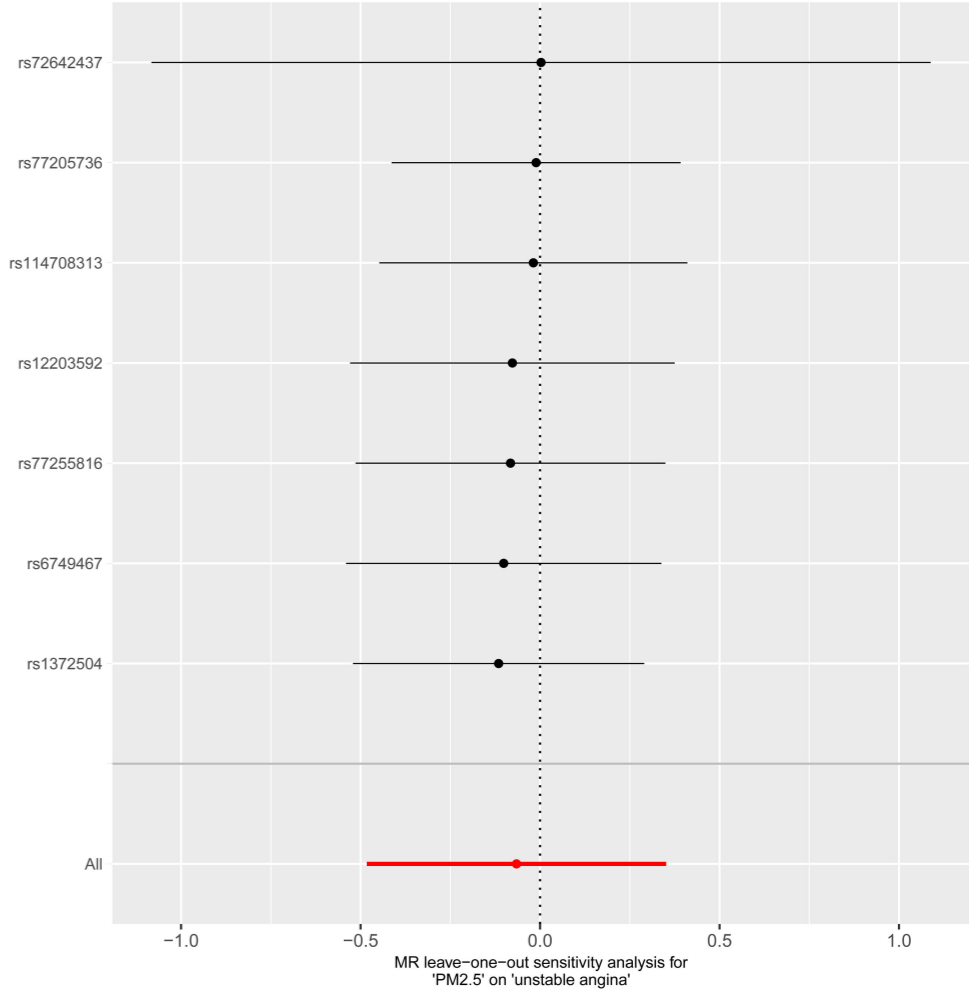

Supplement: Supplementary file 1 [file toxics-13-00021-s001.zip › Figure S1.pdf]

A

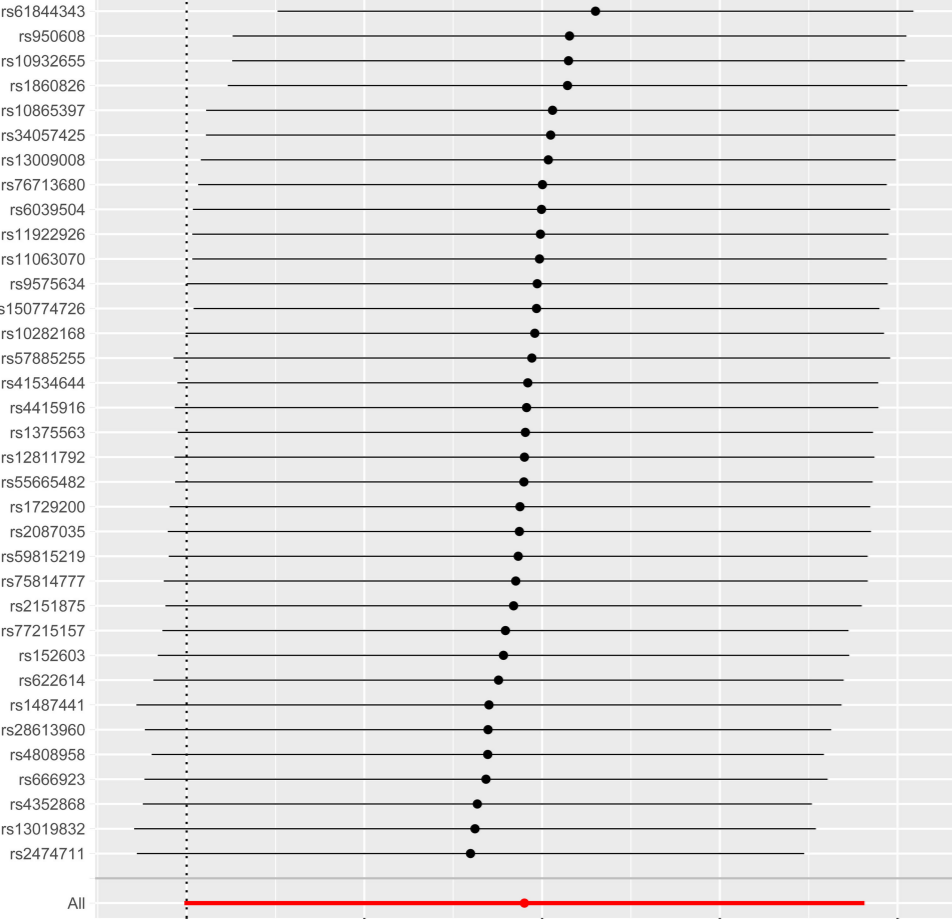

B

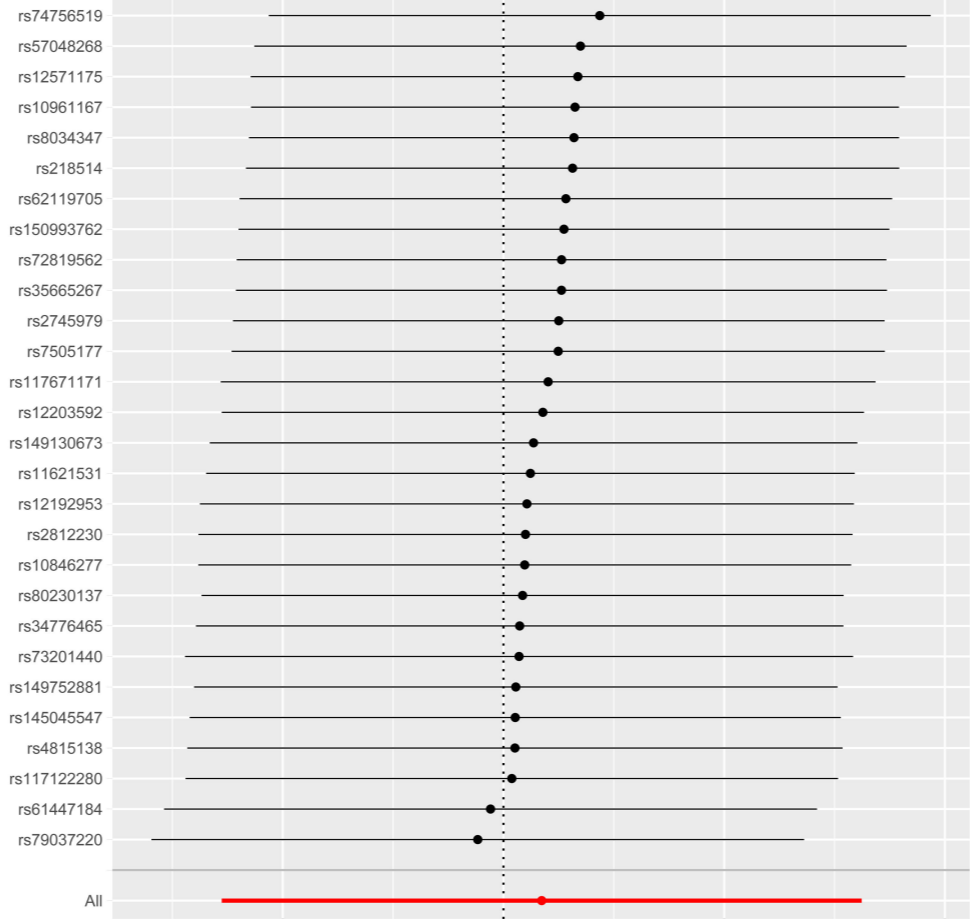

C

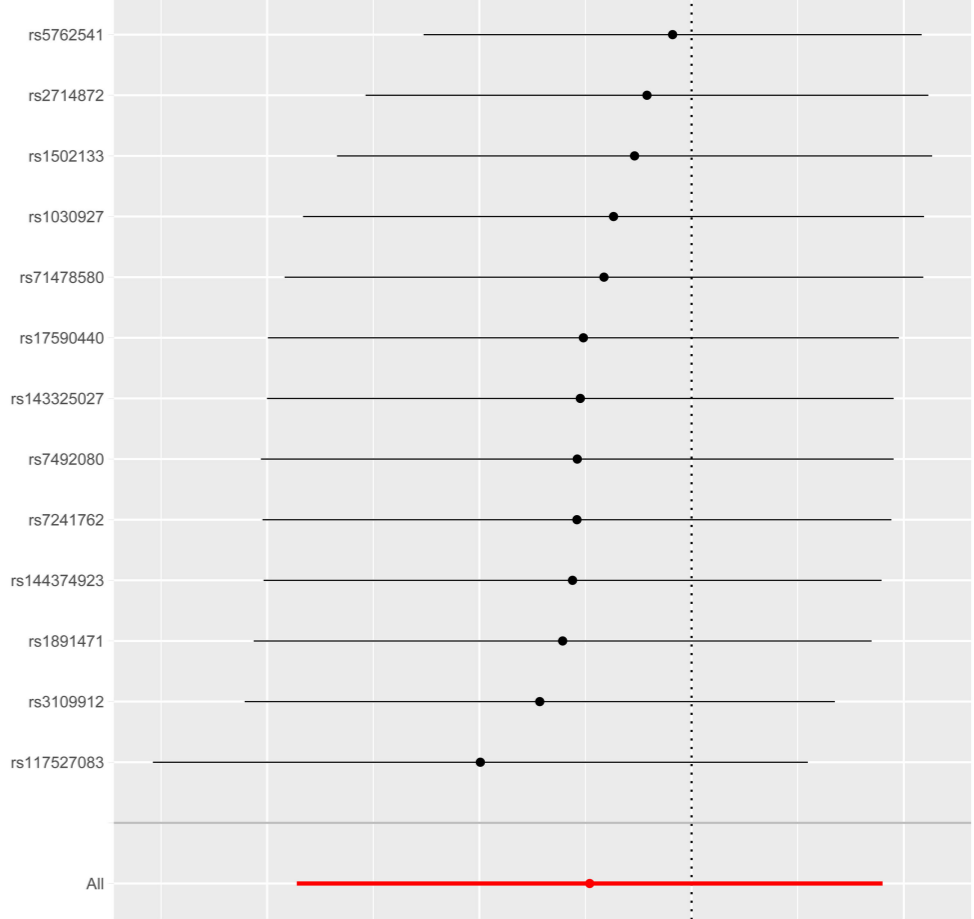

D

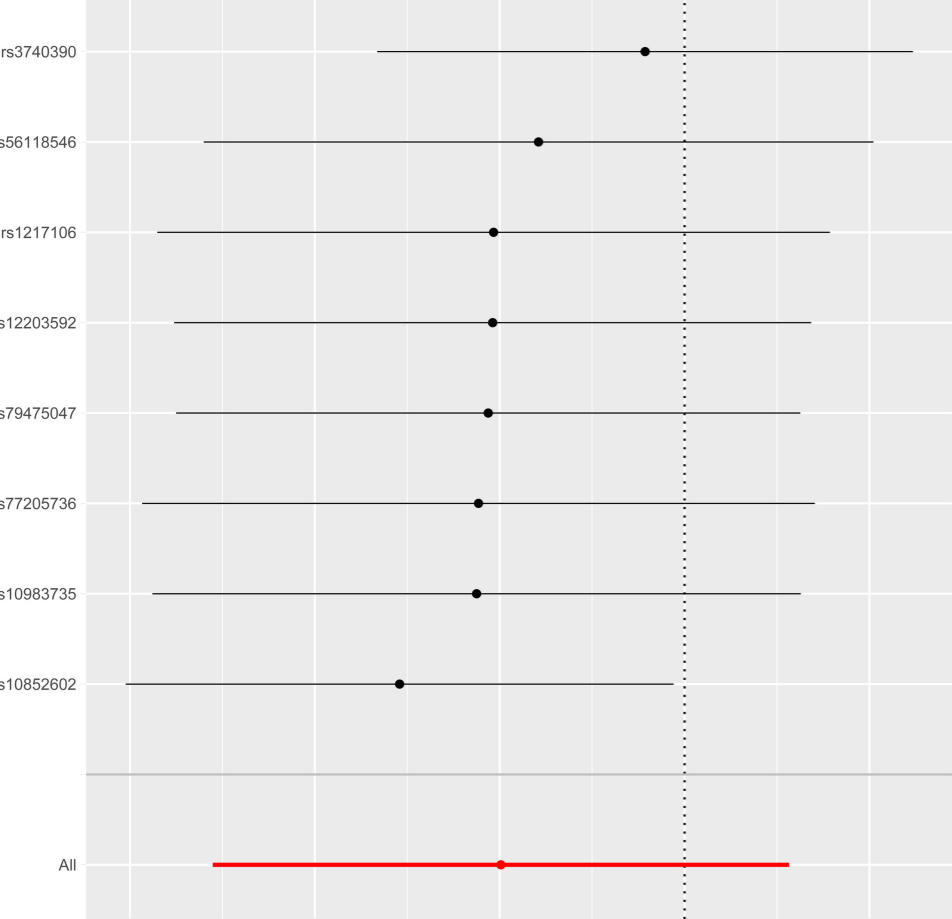

E

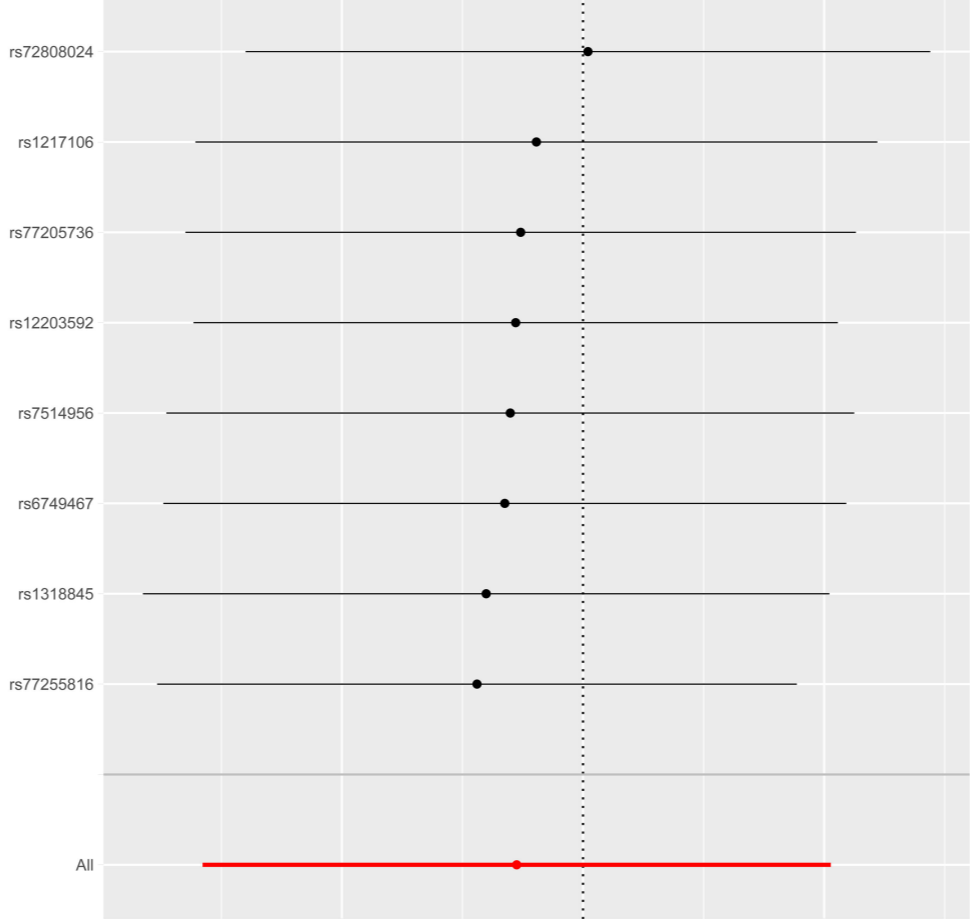

F

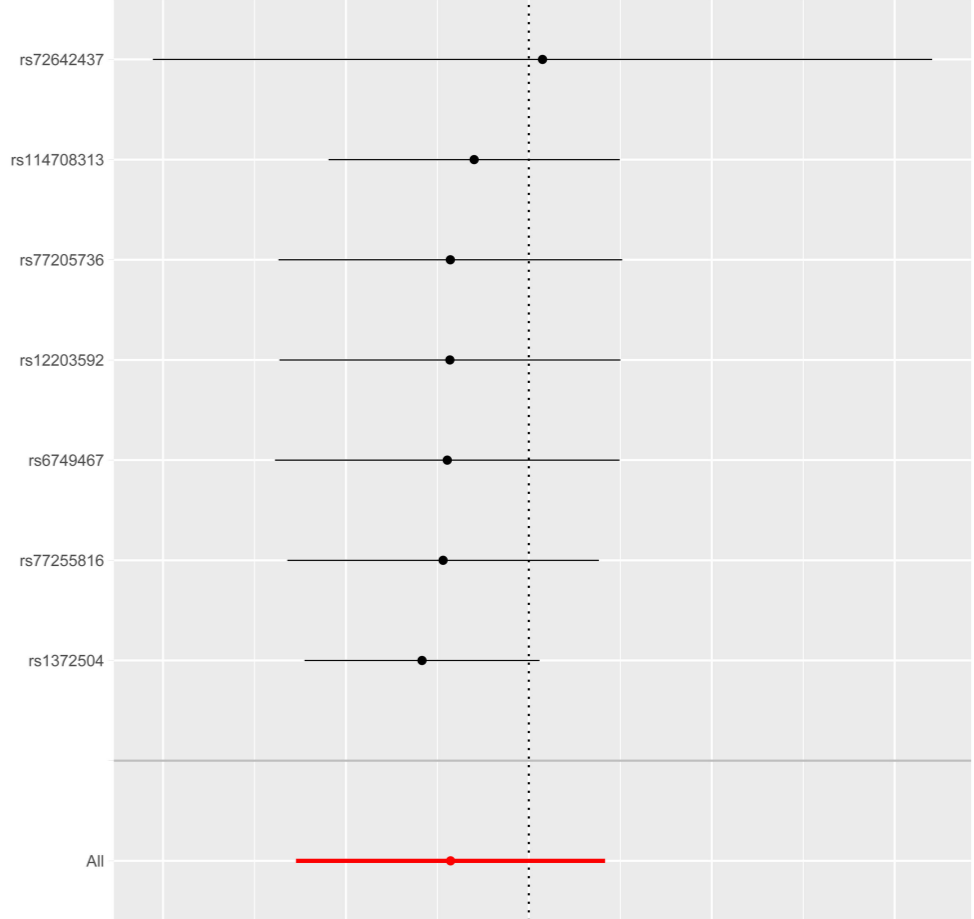

Supplement: Supplementary file 1 [file toxics-13-00021-s001.zip › Figure S2.pdf]
